# Quantification of total T-cell receptor diversity by flow cytometry and spectratyping

**DOI:** 10.1186/1471-2172-14-35

**Published:** 2013-08-06

**Authors:** Stanca M Ciupe, Blythe H Devlin, Mary Louise Markert, Thomas B Kepler

**Affiliations:** 1 Department of Mathematics, Virginia Tech, 460 McBryde Hall, Blacksburg, VA 24060, USA; 2 Department of Pediatrics, Duke University Medical Center, Durham, NC 27710, USA; 3 Department of Immunology, Duke University Medical Center, Durham, NC 27710, USA; 4 Department of Microbiology, Boston University School of Medicine, Boston MA 02118, USA

## Abstract

**Background:**

T-cell receptor diversity correlates with immune competency and is of particular interest in patients undergoing immune reconstitution. Spectratyping generates data about T-cell receptor CDR3 length distribution for each BV gene but is technically complex. Flow cytometry can also be used to generate data about T-cell receptor BV gene usage, but its utility has not been compared to or tested in combination with spectratyping.

**Results:**

Using flow cytometry and spectratype data, we have defined a divergence metric that quantifies the deviation from normal of T-cell receptor repertoire. We have shown that the sample size is a sensitive parameter in the predicted flow divergence values, but not in the spectratype divergence values. We have derived two ways to correct for the measurement bias using mathematical and statistical approaches and have predicted a lower bound in the number of lymphocytes needed when using the divergence as a substitute for diversity.

**Conclusions:**

Using both flow cytometry and spectratyping of T-cells, we have defined the divergence measure as an indirect measure of T-cell receptor diversity. We have shown the dependence of the divergence measure on the sample size before it can be used to make predictions regarding the diversity of the T-cell receptor repertoire.

## Background

The immune system’s ability to fight a large array of foreign particles is facilitated by the diversity of the T-cell receptor (TCR) repertoire [1]. This diversity is generated during thymocyte development by a process of somatic recombination. Inside the thymus, the constant (C) and variable (V) domains of the *α* and *β* chains of the TCR are assembled via random genetic rearrangements of the variable (V), diversity (D) and joining (J) gene segments [2]. Additional diversity is added through imprecise joining of the V and J regions along with random nucleotide additions and deletions at the V(D)J junctions [2,3]. Consequently, most of the variability lies in the third complementary determining region (CDR3) which is encoded by the V(D)J junction and comes in contact with the antigenic peptide on the surface of peptide/major histocompatibility complex (pMHC) molecules [4,5]. While the total number of lymphocytes in the blood can be directly measured, assessment of the diversity of the TCR repertoire requires more complex and indirect assays in a research setting. Such assays include flow cytometry, spectratyping and nucleotide sequencing.

Different T-cell clones use different V gene families in the rearrangement of their *β* chains. Through the use of commercially available monoclonal antibodies (named TCR V *β*), one can use standard flow cytometry on whole blood samples to determine the percentage of CD4 T-cells that use a given TCR BV family in subjects or controls. Measures of heterogeneity of TCR BV family usage in these CD4 T-cells can be used as a substitute for TCR repertoire diversity [6]. Flow cytometry is not only faster, cheaper, and technically simpler to use; the data reflects real population percentages.

Spectratyping uses messenger RNA (mRNA) from T-cells to amplify, by PCR, the complementary DNA (cDNA) across the CDR3 region. This generates information about the heterogeneity of the relative frequencies of different CDR3 length products within a functional TCR BV family. Because different T-cell clones have different sequences or lengths of CDR3, analysis of the CDR3 length distributions can be used to determine the overall TCR repertoire diversity [7-11]. Spectratyping has the advantage of providing a finer level of resolution than just analyzing BV gene family expression on the T-cells of flow cytometry. Although spectratyping provides the total number of CDR3 sizes and their pattern of distribution, the investigator cannot determine the frequency of cells used by a particular BV family. Amplifications of variations from a background distribution of each individual BV family may lead to over-representation of immunodominant clonotypes and therefore yield results that are not representative of the contribution of those cells in the entire T-cell repertoire.

TCR diversity can also be assessed by nucleotide sequencing of DNA CDR3 regions, but this is labor-intensive and generates an even lower level of resolution of the whole T-cell repertoire compared to spectratyping [12].

This paper focuses on the role of flow cytometry in measuring T-cell population diversity and compares it to T-cell population diversity as given by spectratyping. Traditionally, spectratyping data is quantified using a wide range of methods from visual [13,14] to quantitative scoring [15-17]. Our group previously described the use of a likelihood method for measuring deviation from a normal TCR repertoire [9,11]. For each observed CDR3 length distribution by spectratyping, we compute the Kullback-Leibler divergences between the patient CDR3 length distribution and a known reference distribution [9,11]. We have modified the Kullback-Leibler divergence to measure the deviation of T-cell receptor diversity from normal. This was done by accounting for both the TCR BV family usage as measured by flow cytometry and by comparing the utility of this method to CDR3 length distribution as measured by spectratyping [11].

Estimator bias is a concern when using this method of divergence scoring. In particular, it is desirable to determine how much deviation in the computation of the divergence occurs when the initial number of lymphocytes used in generating the data is varied. We have addressed this question in the context of divergence measures generated individually by flow cytometry and spectratyping. The results are especially useful when using the techniques for limited numbers of cells.

## Results

We used the Kullback-Leibler divergence to quantify similarities between different frequency distributions in the T-cell repertoire diversity when measured by either flow cytometry or spectratyping. We started with two assumptions: 1) the reference distribution corresponds to a polyclonal TCR repertoire and 2) in individual subjects, a positive divergence determines the deviation from the normal TCR repertoire. The flow divergence, *D*_*f*_, is the distance between the individual and the perfectly sampled reference control distributions of all TCR BV family usage measured by flow cytometry. The spectratype divergence, *D*_*s*_, is the distance between the individual and the perfectly sampled reference control distributions of the CDR3 lengths of each TCR BV family and averaged over all TCR BV families as measured by spectratyping (see section Kullback-Leibler divergence and [9]).

**Figure 1:**
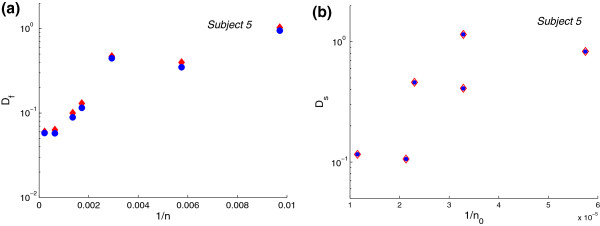
**Measured and corrected divergence measures as function of inverted sample number. (a)** Measured flow divergence, *D*_*f*_, (red solid diamonds) and corrected flow divergence, *D*_*f*,corr_, (blue circles) as functions of the inverted sample number 1/*n*; **(b)** Measured spectratype divergence, *D*_*s*_, (red empty diamonds) and corrected spectratype divergence, *D*_*s*,corr_, (blue circles) as a function of the inverted sample number 1/*n*_0_ in one DiGeorge patient.

**Figure 2:**
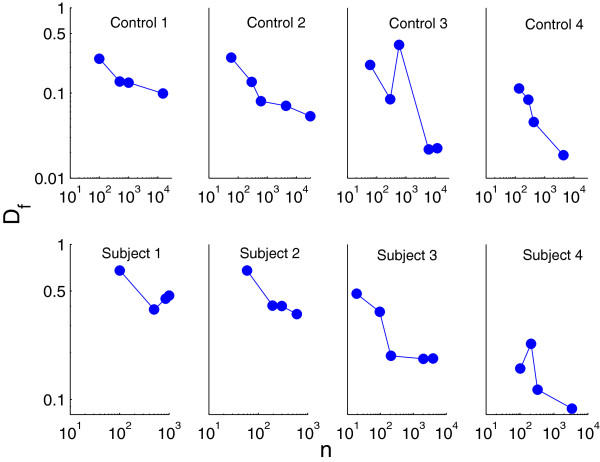
**Flow divergence, *D***_***f***_**, as a function of sample size n (∙), presented on a log-log scale.**

We specifically wanted to assess the performance of the divergences *D*_*f*_ and *D*_*s*_ in predicting the diversity of the T-cell receptor repertoire under stressful, i.e. cell limited, circumstances. While *D*_*f*_ and *D*_*s*_ account for deviations from normal of distributions of TCR BV family usage and CDR3 lengths within each TCR BV family, additional variability is added due to the dependence on the number of measured events, *n*, for every individual patient/control (see Figures 1 and 2). Knowing the sample size *n* and the dimensions of the measured space, *L*_*i*_, we derived the corrected divergence value, *D*_*i*,corr_ (see section ‘Sampling bias - theoretical derivation’) to be given by

(1) $$D_{i,\text{corr}}=D_{i}-\frac{L_{i}-1}{2n},$$

where *i* = *f*,*s* for flow cytometry and spectratyping, respectively. *L*_*f*_ is the number of BV families used in the flow cytometry assay (in our case 18) and *L*_*s*_ is the number of CDR3 lengths used in the spectratype assay (in our case 14).

Therefore, only the number of measured events, *n*, and the dimension of the measured space, *L*_*i*_ are needed to correct the divergence measures. We used this formula to assess the performance of *D*_*f*_ and *D*_*s*_ measures in an athymic DiGeorge subject (Figure 1) during a period of limited numbers of peripheral blood T-cells as the patient underwent immune reconstitution following thymus transplantation.

### Flow cytometry results

Flow divergence measurements, *D*_*f*_, were determined at seven time points following thymus transplantation in DiGeorge subject 5 (Table 1). For each time point, the number of CD4 T-cell was known (Table 1). The corrected divergence *D*_*f*,corr_ is found by subtracting (*L*_*f*_ - 1)/2*n*, where *L*_*f*_ = 18, from the measured divergence *D*_*f*_ at each time point (Table 1). The measured and corrected divergences as a function of 1/*n* are plotted in Figure 1(a). When we use samples with low event numbers, we noted an overestimate in the measured *D*_*f*_ compared to *D*_*f*_ estimates from samples with high event numbers, for which the correction is not significant. Formula (1) helped address the effect of event number on the *D*_*f*_ prediction.

**Table 1:** **Average CD4 T-cell sample size, measured flow divergence *D***_***f***_**, and corrected flow divergence *D***_***f*, corr **_**in a DiGeorge subject**

**Days after**	**Average CD4 nr**	**Measured flow**	**Corrected flow**
**transplant**	**in gate (*n*)**	***D***_***f***_**value**	***D***_***f*, corr**_**value**
70	341	0.47	0.44
88	103	1.02	0.94
117	174	0.39	0.34
145	581	0.129	0.11
181	737	0.103	0.091
398	1569	0.063	0.057
868	4514	0.06	0.058

Values are measured over time following thymic transplantation.

To further test the dependence of *D*_*f*_ on the sample size we assumed that *D*_*f*_ is a function of the decreasing event numbers in the CD4 T-cell gate used for TCR BV analysis. For this analysis we used a single blood sample collection from each of four complete DiGeorge subjects after thymus transplantation and from each of four healthy controls. Each blood sample was serially diluted, followed by flow cytometry. The results are presented in Table 2 and the plot of *D*_*f*_ as a function of *n* is presented in Figure 2.

**Table 2:** **Summary of T-cell sample size and the corresponding flow divergence values *D***_***f***_

***Subject***	**Average CD4 T-cell nr**	**Measured flow**
	**in gate n**	**divergence *D***_***f***_
Control 1	66	0.252
	340	0.135
	675	0.132
	10051	0.098
Control 2	58	0.260
	290	0.135
	603	0.079
	4438	0.070
	29438	0.053
Control 3	60	0.214
	290	0.084
	585	0.366
	5965	0.021
	11889	0.022
Control 4	136	0.112
	282	0.083
	425	0.045
	4354	0.018
Subject 1	89	0.679
	445	0.379
	756	0.445
	887	0.466
Subject 2	59	0.678
	194	0.403
	299	0.399
	605	0.355
Subject 3	19	0.479
	95	0.366
	207	0.191
	2013	0.182
	3946	0.183
Subject 4	103	0.158
	213	0.229
	329	0.115
	3367	0.087

For each of these eight cases, we wanted to predict the corrected divergence value, *D*_*f*,corr_, using the measured *D*_*f*_s and determine their dependence on the sample size *n*. We define a three parameter linear model given by

(2) y(n)=α+C/n+ε,

where, *y*(*n*) is the observed *D*_*f*_ and *n* is the number of CD4 T-cells in the sample. The intercept *α* is the true divergence, *D*_*f*,corr_, and the slope *C* quantifies the rate at which the diversity is dependent on the sample size. In equation (1), slope *C* corresponds to the (*L*_*f*_ - 1)/2 value, which for an assay that uses 18 BV families, reduces to 8.5. The errors, *ε*, are independent and normally distributed.

**Figure 3:**
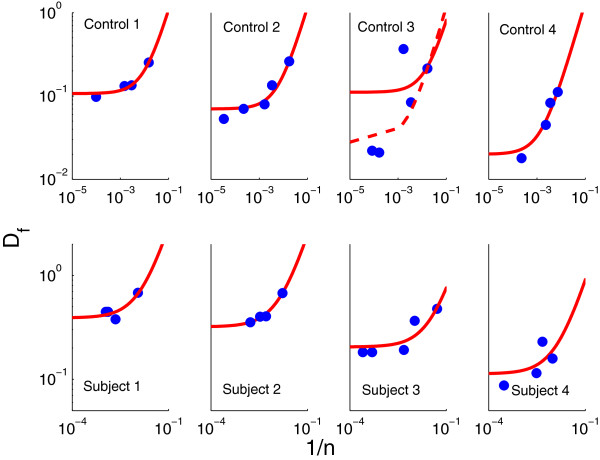
**Flow divergence *D***_***f***_** as a function of the inverted sample number *1/n* in eight subjects. **The solid line represents the fit of the three parameter linear model (2) to the data (∙). Results are presented on a log-log scale. The same model was fitted to a data set that excluded point (0.0017,0.366) for control 3 (dashed line). The best parameter estimates and their 90% confidence intervals are presented in Table 3.

We derived estimates and 95% confidence intervals for parameters *α* and *C* for each of eight individuals by fitting *y*(*n*), as given by (2), to the measured *D*_*f*_ values in Table 1 for CD4 T-cell numbers *n*. For the fitting routine we used a descent method for univariate functions [18]. The parameter values and their confidence intervals are presented in Table 3. The regression curves and data are presented in Figure 3.

**Table 3:** **Parameter values and confidence intervals for model** (2)

***Subject***		**Value**	**CI**
Control 1	*α*	0.107	[0.079,0.135]
	C	9.7	[6.1, 13.4]
Control 2	*α*	0.07	[0.02,0.129]
	C	10.9	[4.7, 17.2]
Control 3	*α*	0.111	[-0.17,0.373]
	C	6.9	[-29, 43]
Control 4	*α*	0.02	[-0.027,0.067]
	C	13	[2, 24]
Subject 1	*α*	0.39	[0.214, 0.574]
	C	25	[-6.3, 56]
Subject 2	*α*	0.32	[0.253, 0.377]
	C	21.3	[14.5, 28.1]
Subject 3	*α*	0.205	[0.087, 0.322]
	C	5.5	[0.7, 10.4]
Subject 4	*α*	0.113	[-0.116, 0.342]
	C	7.9	[-33, 49]

Moreover, if we consider the slope *C* to be equal among the subjects we can simultaneously fit the following model to the data from all subjects.

(3) $$y_{i}(n)=\alpha_{i}+C/n+\varepsilon_{i},$$

where *α*_*i*_ are the corrected divergence values for the patient *i*, with *i* = 1,...,8. The rate at which the diversity is dependent on the sample size, *C*, is considered constant among the subjects. The errors for each of the subjects, *ε*_*i*_, are independent and normally distributed.

**Figure 4:**
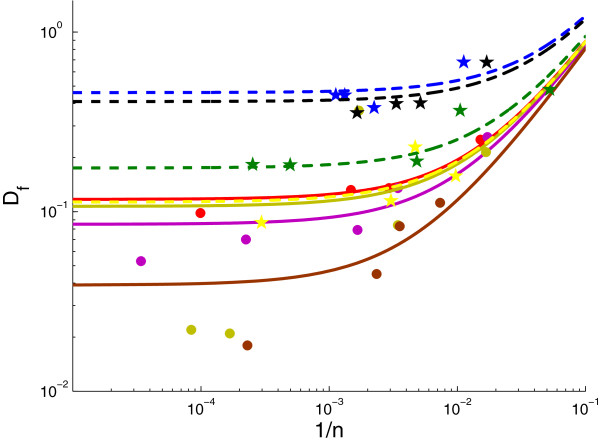
**Flow divergence *D***_***f***_** as a function of the inverted sample number *1/n* for the same slope *C*. **The solid and dashed lines shows the fit of a three parameter linear model (3) to the data (∙). The results are presented on a log-log scale. The best parameter estimates and their 90% confidence intervals are presented in Table 4.

The fitting procedure was done using a quasi-Newton method for finding the minimum of a multivariate function [18]. The predicted parameter values and their confidence intervals are presented in Table 4. The regression curves and data are presented in Figure 4.

**Table 4:** **Parameter values and confidence intervals for model** (3)

***Subject***	***α***	**CI**
Control 1	0.117	[0.033,0.202]
Control 2	0.085	[0.009,0.161]
Control 3	0.107	[0.032,0.184]
Control 4	0.039	[-0.045,0.123]
Subject 1	0.46	[0.38, 0.55]
Subject 2	0.41	[0.32, 0.49]
Subject 3	0.175	[0.089, 0.261]
Subject 4	0.113	[0.029, 0.2]
*Subject*	*C*	CI
All	7.705	[4.55, 10.85]

#### ***Events estimation***

From the flow cytometry analysis we can estimate the minimum number of CD4 T-cells needed in a sample for an accurate *D*_*f*,corr_ estimate. If we want our estimates to be 90% accurate, *i.e.*, err = 0.1, then the ratio between the corrected and measured divergence has to be less than err,

(4) $$\frac{1/\text{nC}}{\alpha +1/\text{nC}}<\text{err}.$$

This translates into the following condition

(5) $$n>\frac{C(1-\text{err})}{\text{err}\times \alpha }.$$

From our estimates *C* = 7.705 and *α* = 0.19 (median 0.12). This implies the sample size, *n*, must be larger than 364 (median 577) cells for an accurate *D*_*f*,corr_ estimate. In our case, we gated the flow cytometry on CD4 T-cells, so more than 364 CD4 T-cells, or events, must be captured in the flow analysis.

### Spectratype results

Spectratype divergence measurements, *D*_*s*_, were determined in five patients for three to seven time points following thymic transplantation (Table 5). For each time point, the number of CD3 T-cell used to isolate RNA, *n*_0_, is known (Table 5). Starting with a fixed amount of RNA, complementary DNA (cDNA) is generated in a reverse transcriptase reaction and used with each of *π* = 23 different primers to amplify the CDR3 region from each BV gene.

**Table 5:** **CD3 T-cell sample size, measured spectratype divergence *D***_***s***_**, and corrected spectratype divergence *D***_***s,,corr***_** in a DiGeorge subject**

***Subject***	**Days after transplant**	**CD3 T-cells *n***_***0***_	**Measured *D***_***s***_**value**	**Corrected *D***_***s*, corr**_**value**
Subject 1	9	420,000	0.91	0.9096
	34	12,220,000	0.61	0.61
	70	550,000	0.97	0.9697
Subject 4	540	670,000	0.039	0.0388
	1540	1,260,000	0.073	0.0729
	2017	1,140,000	0.076	0.0759
Subject 5	70	700,000	1.15	1.1498
	88	400,000	0.83	0.8296
	117	700,000	0.41	0.4098
	145	1,000,000	0.46	0.4599
	181	1,080,000	0.106	0.1059
	398	2,000,000	0.116	0.1159
Subject 6	175	1,440,000	0.107	0.1069
	209	800,000	0.168	0.1678
	286	1,480,000	0.086	0.0859
	730	1,200,000	0.12	0.1199
Subject 7	102	380,000	0.43	0.4296
	130	460,000	0.23	0.2297
	166	500,000	0.08	0.0797
	372	1,250,000	0.14	0.1399

Values are measured over time following thymic transplantation.

The corrected *D*_*s*,corr_ is found by subtracting (*L*_*s*_ - 1)/2*n*, where *n* = *n*_0_/*π*, from the measured divergence at each time point, where *L*_*s*_ = 14 (Table 5). The measured and corrected divergences as a function of 1/*n*_0_ are plotted in Figure 1(b). We note that there is no correction in the measured spectratype divergence, *D*_*s*_, since the number *n*_0_ of CD3 T-cells that we are starting with is always high.

### Total divergence

By combining the individual contributions of flow and spectratype divergence, we defined the total divergence, *D* (see section ‘Kullback-Leibler divergence’). *D* measures the divergence of the individual from the perfectly sampled reference control and accounts for differences in distributions of CDR3 lengths within each TCR BV family by spectratyping as well as differences in distributions of overall TCR BV families by flow cytometry. Corrections in the flow and spectratype divergences are sufficient to ensure that the total divergence is independent of the sample size.

## Discussion

The data used in our study came from flow cytometry and spectratype assays in both DiGeorge subjects after thymus transplantation and healthy adult volunteers. This study presents significant information regarding the utility of flow cytometry, as well as spectratyping, to assess the diversity of the antigen receptor repertoire. Importantly, these data identify a bias in measurement errors which must be corrected. The paper presents the relationships between the number of gated events in the flow cytometry assay, as well as the number of CD3 T-cells in the spectratype assay, and the information-theory measures, *D*_*f*_ and *D*_*s*_, used as surrogates of TCR diversity.

We addressed a critical issue of estimator bias. Starting with the assumption that such a bias exists, we have derived ways to account for the error in the measured divergences. We show that *D*_*f*_ and *D*_*s*_ can be corrected by substracting a number inversely proportional to the sample size.

For the flow cytometry data, the constant of proportionality can either be deduced theoretically as a function of the total number of BV TCR families used in the flow cytometry assay, or derived from a statistical model applied to individual data. Both methods predict similar results, with the constant equal to 8.5 in the theoretical approach and 7.7 in the statistical approach. It is important to note that we found a direct correlation between the measured *D*_*f*_ and the sample size in five out of eight subjects (see Table 6).

**Table 6:** Correlation coefficient and p-values as given by a Pearson comparison test, between the inverse average number of CD4 T-cell used in flow cytometry assays and the flow divergence

***Subject***	**Correlation coefficient**	**p-value**
Control 1	0.99	0.0076
Control 2	0.98	0.0031
Control 3	0.32	0.58
Control 4	0.96	0.035
Subject 1	0.92	0.075
Subject 2	0.99	0.005
Subject 3	0.9	0.036
Subject 4	0.5	0.49

Our study allows us to predict a lower bound for the number of CD4 T-cells needed in the flow cytometry gated events. We have shown that at least 364 CD4 T-cells have to be counted as gated events for a 90% confidence in the *D*_*f*_ measures. With fewer gated events, the *D*_*f*_ measurement cannot be used as a substitute for diversity. This is particularly important to keep in mind when assessing patients with limited numbers of T-cells, such as those undergoing immune reconstitution following thymus, stem cell or bone marrow transplantation. Each of these is a clinical situation in which the development of the T-cell repertoire correlates to immune competency. Thus, these data provide a quantitative basis by which T-cell repertoire diversity can be assessed by flow cytometry.

For the spectratype data, the results are quite different. Although, using the same theoretical approach, we derive a constant, *C* = 6.5, that accounts for measurement bias; thus, the corrected spectratype divergence is identical to the observed divergence. Moreover, we find no correlation between the measured spectratype divergence, *D*_*s*_, and the sample size in four out of five patients (Table 7).

**Table 7:** Correlation coefficient and p-values as given by a Pearson comparison test, between the inverse total number of CD3 T-cell used in spectratype assays and the spectratye divergence

***Subject***	**Correlation coefficient**	**p-value**
Subject 1	0.92	0.25
Subject 4	-0.98	0.11
Subject 5	0.66	0.15
Subject 6	0.97	0.03
Subject 7	0.64	0.35

The total divergence actively incorporates the flow divergence. Correction in the flow divergence, *D*_*f*_, guarantees independence of the total divergence, *D*, from the sample size.

## Conclusions

In conclusion, sample size is a sensitive parameter in the predicted flow divergence values, but not in the spectratype divergence values. Although using flow cytometry to assess T-cell repertoire diversity is a valuable tool, one must have sufficient cells, or events, in the flow cytometry gate before using either the flow or the total divergence as a prediction for the TCR repertoire diversity.

## Methods

### Human subjects

Blood samples used in our study come from healthy adult controls and from infants with complete DiGeorge anomaly after thymus transplantation [19]. Blood was obtained under protocols approved by Duke University Medical Center Internal Review Board (IRB). T-cell repertoire evaluation was done by flow cytometry. Whole blood samples were evaluated using 22 monoclonal antibodies directed against CD4 and a total of 18 TCR BV families (Beckman Coulter and BD Biosciences - see Tables 8 and 9).

**Table 8:** List of TCR BV families and antibodies used in the flow cytometry assay

**Antibody names**	**Clone**	**Family name**^∗^
V *β*1	BL37.2	TRBV9
V *β*2	MPB2D5	TRBV20
V *β*3	CH92	TRBV28
V *β*4	WJF24	TRBV29
V *β*5.1	IMMU157	TRBV5
V *β*5.3	3D11	TRBV5
V *β*5.2	36213	TRBV5
V *β*7.1	ZOE	TRBV4
V *β*7.2	Zizou4	TRBV4
V *β*8.1 & V *β*8.2	56C5	TRBV12
V *β*9	FIN9	TRBV3
V *β*11	C21	TRBV25
V *β*12	VER2.32.1	TRBV10
V *β*13.2	H132	TRBV6
V *β*13.6	JU-74	TRBV6
V *β*14	CAS1.1.3	TRBV27
V *β*16	TAMAYA 1.2	TRBV14
V *β*17	E17.5F3	TRBV19
V *β*18	BA62	TRBV18
V *β*20	ELL 1.4	TRBV30
V *β*22	IMMU 546	TRBV2
V *β*23	AF23	TRBV13

The antibodies were purchased from Immunotech (Beckman Coulter) and used for the analysis. A kit IOTest Beta Mark became available during the study and was used in place of individually purchased antibodies. ^∗^Nomenclature of the IMGT, the international ImMunoGeneTics information system http://www.imgt.org.

**Table 9:** List of TCR VB families and antibodies excluded from the flow cytometry studies

**Antibody names**	**Clone**	**Family name**^∗^
V *β*13.1 & 13.4 & 13.6	IMMU 222	TRBV6-5 & 6-6 & 6-9
V *β*21.3	IG125	TRBV11-2

These antibodies are included in the kit but were not included in the analysis. ^∗^Nomenclature of the IMGT, the international ImMunoGeneTics information system http://imgt.cines.fr.

### Human subjects

Subjects were enrolled in protocols that were approved by the Duke University Health System Institutional Review Board and were reviewed by the Food and Drug Administration under an Investigational New Drug application. All subjects were children. The parent(s) of each subject provided written informed consent.

### Flow cytometry

Reference distributions of TCR BV family usage determined by flow cytometry were generated from peripheral blood samples of fifty healthy individuals (see Table 10). Similar distributions of TCR BV usage were derived from four additional controls and four DiGeorge subjects [19] who underwent thymus transplantation.

**Table 10:** Mean % of CD4 T-cells that use a TCR BV family as predicted by the flow cytometry assay

**Antibody names**	**Mean % of CD4 T-cells**
V *β*1	3.21
V *β*2	9.79
V *β*3	4.80
V *β*4	2.58
V *β*5.1	6.78
V *β*5.3	0.97
V *β*5.2	0.70
V *β*7.1	1.89
V *β*7.2	1.12
V *β*8.1 & V *β*8.2	4.71
V *β*9	3.48
V *β*11	0.73
V *β*12	1.85
V *β*13.2	2.66
V *β*13.6	1.84
V *β*14	3.03
V *β*16	0.91
V *β*17	5.79
V *β*18	1.96
V *β*20	2.35
V *β*22	4.12
V *β*23	0.45

Note that the antibody used in flow cytometry assay covers approximately 70% of CD4 T-cells. The values are averaged across 50 normal volunteers.

### Spectratyping

**Figure 5:**
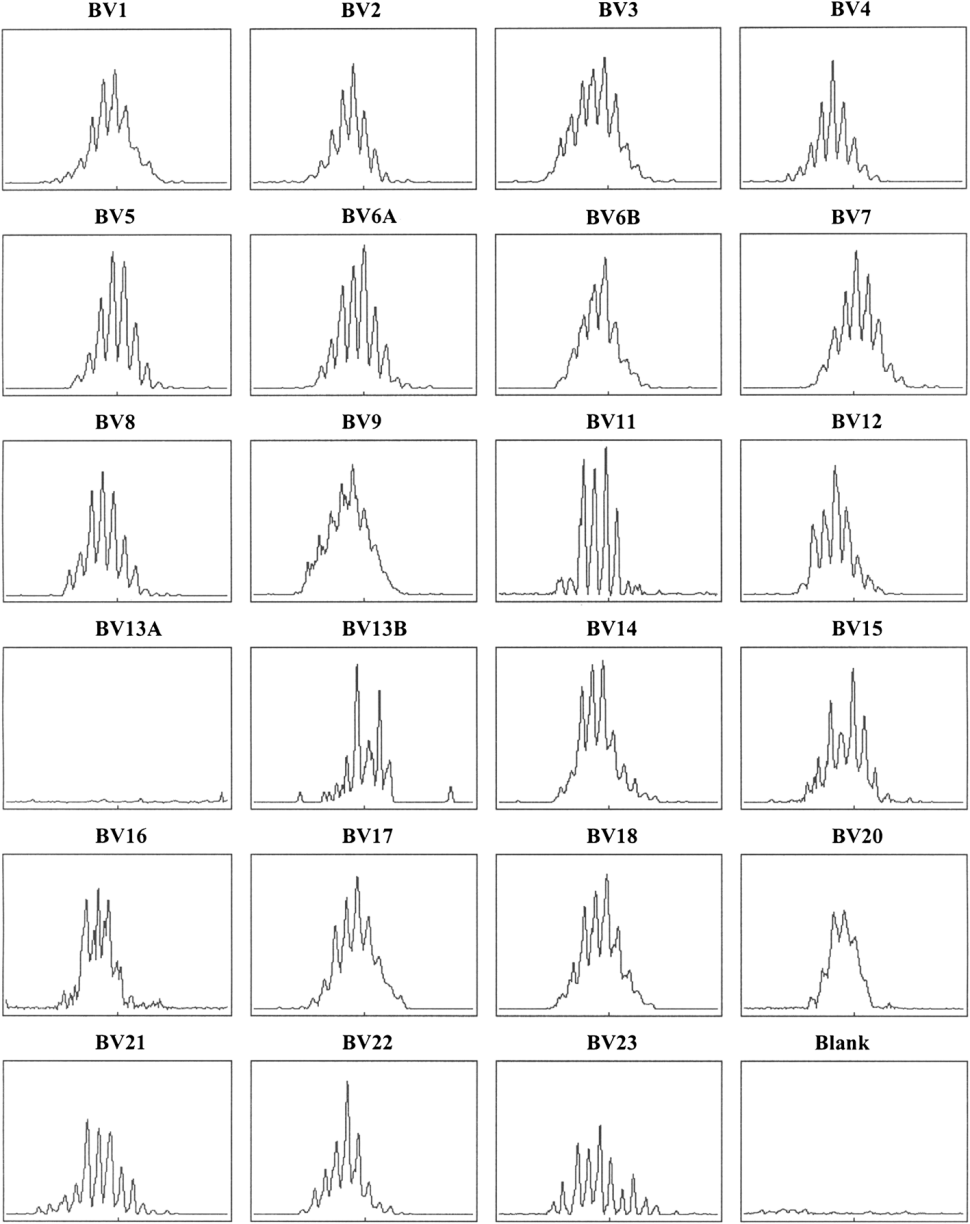
**CD4 T-cell spectratype data. **Spectratype histograms show the number of CD4 T-cells bearing receptors versus CDR3 length for each TCR BV families tested.

CD3 T-cells from the peripheral blood of patients were isolated. RNA was prepared and used for cDNA synthesis. The cDNA was used as a template for 23 TCR BV specific primer pairs to amplify the complete CDR3 region by PCR [10]. Each PCR product, representing a different TCR BV family, was size separated by electrophoresis and the product lengths were identified using the GeneScan software (Applied Biosciences). An example of spectratype data in a healthy adult is presented in Figure 5, which shows the histograms of the number of CD4 T-cells versus CDR3 length for each TCR BV family.

### Kullback-Leibler divergence

#### ***Flow Kullback-Leibler divergence***

Let *P* = {*P*_*i*_,*i* = 1,...,*n*_*F*_} be the relative frequencies corresponding to the ideal, perfectly sampled reference distribution of BV family *i* usage, where *n*_*F*_ is the number of BV families (in our case 18). Let *P* = {*P*_*i*_,*i* = 1,...,*n*_*F*_} be the relative frequency of cells that use BV family *i* in individual control/subjects. The null hypothesis is that a normal polyclonal TCR repertoire has a distribution identical with that of the reference distribution. Deviation from the normal repertoire seen in subjects can be quantified by the flow Kullback-Leibler divergence [9]

(6) $$D_{f}=\overset{n_{F}}{\underset{i=1}{\sum }}p_{i}\log\frac{p_{i}}{P_{i}}.$$

The flow Kullback-Leibler divergence is a measure of the distance between the two frequency distributions or, equivalently, it is the inefficiency of assuming that the distribution of BV family usage is *p*_*i*_, *i* = 1,...,*n*_*F*_, when the true frequency usage is *P*_*i*_,*i* = 1,...,*n*_*F*_.

#### ***Spectratype Kullback-Leibler divergence***

Similarly, let *p* = {*p*_*ij*_ = *q*_*i*_*r*_*j*/*i*_,*i* = 1,...,*n*_*F*_ and *j* = 1,...,*n*_*C*_}, and *P* = {*P*_*ij*_ = *Q*_*i*_*R*_*j*/*i*_,*i* = 1,...*n*_*F*_ and *j* = 1,...*n*_*C*_}, respectively, be the relative numbers of T-cells of CDR3 lengths *j*, given that the BV family *i* is used in individual patient/controls and reference controls as determined by spectratype. Here *n*_*C*_ is the number of CDR3 lengths (in our case 14), (*q*,*Q*)_*i*_ are the relative frequencies of cells which use the BV family *i* and (*r*,*R*)_*j*/*i*_ the relative frequencies of of cells that have CDR3 length *j*, given that they use the BV family *i*. The null hypothesis is that a normal polyclonal TCR repertoire has a distribution of CDR3 lengths identical with that of the reference distribution. Deviation of from the normal repertoire, as seen in patients, can be quantified by the spectratype divergence for each TCR BV family *i* as follows

(7) $$D_{s/i}=\overset{n_{C}}{\underset{i=1}{\sum }}r_{j/i}\log\frac{r_{j/i}}{R_{j/i}},$$

and the total spectratype divergence, which is the average of spectratype divergences of TCR BV families *i*, *i*∈{1,...,*n*_*F*_} is given by

(8) $$D_{s}=\frac{1}{n_{F}}\overset{n_{F}}{\underset{i=1}{\sum }}D_{\text{KL},\text{spec}/i}.$$

#### ***Total Kullback-Leibler divergence***

We can combine these two measures to obtain a total divergence measure from normal repertoire, derived as follows

(9) $$\begin{array}{lll} D & =\overset{n_{F}}{\underset{i=1}{\sum }}\overset{n_{C}}{\underset{j=1}{\sum }}p_{\text{ij}}\log\frac{p_{\text{ij}}}{P_{\text{ij}}}=\overset{n_{F}}{\underset{i=1}{\sum }}\overset{n_{C}}{\underset{j=1}{\sum }}q_{i}r_{j/i}\log\frac{q_{i}r_{j/i}}{Q_{i}R_{j/i}}\;\\ & =\overset{n_{F}}{\underset{i=1}{\sum }}q_{i}\log\frac{q_{i}}{Q_{i}}+\overset{n_{F}}{\underset{i=1}{\sum }}q_{i}\overset{n_{C}}{\underset{j=1}{\sum }}r_{j/i}\log\frac{r_{j/i}}{R_{j/i}}\; & \;\\ & =D_{f}+\overset{n_{F}}{\underset{i=1}{\sum }}q_{i}D_{s/i},\; & \; \end{array}$$

### Sampling bias - theoretical derivation

The distribution of BV family usage (CDR3 length within a BV family) of a perfectly sampled reference control can be described by a *L*_*f*_ (*L*_*s*_)-dimensional multinomial distribution with the parameter vector P, where *p*_*i*_ is the relative numbers of T-cells that use the BV family (CDR3 length) *i*. The distribution of the actual, but not yet observed, BV family (CDR3 length) usage in individual patient/controls are subsamples *q* of the ideal distribution, where *q*_*i*_ are the relative numbers of T-cells that use the BV family (CDR3 length) *i*. The distance between these two distributions is given by the parameter *d*^-1^, with a large *d* accounting for a closer similarity between *P* and *q*. Finally, the observed distribution of BV family usage (CDR3 length), *p*, are samples of *n* measured events for every individual patient/control, where *p*_*i*_ are the relative numbers of T-cells that use the BV family (CDR3 length) *i*. Here *L*_*f*_ (*L*_*s*_) is the dimension of the measured space, *i.e.* the number of BV families used in the flow cytometry assay, in our case 18 (the number of CDR3 lengths used in spectratyping assay, in our case 14).

For a large sampling number, *n*, we can consider the relative frequencies *P*, *q* and *p* to be continuous variables and define their probability distribution functions, pdf, as

(10) $$f(p|P,n,d^{-1})=\int f(p|q,n)f(q|P,d^{-1})d^{L_{i}}q$$

where *i* = *f*,*s*. The pdf of *p*, for *np*_*i*_ large enough, can be approximated using Stirling’s formula (see [9] for a complete computation). Therefore,

(11) $$\begin{array}{c} f(p|q,n)\;=\;n^{L_{i}-1}\frac{\Gamma (n+1)}{\Gamma (np_{i}+1)}\overset{L_{i}}{\underset{i=1}{\prod }}q_{i}^{p_{i}n}\\ \;\approx \frac{n^{(L_{i}-1)/2}e^{-\text{nD}(p|q)}}{\sqrt{{\left(2\pi \right)}^{L_{i}-1}\overset{L_{i}}{\underset{i=1}{\prod }}p_{i}}}\delta (\underset{i}{\sum }p_{i}-1), \end{array}$$

where *δ* is the Dirac delta function and

(12) $$D(p|q)=\overset{L_{i}}{\underset{i=1}{\sum }}p_{i}\log\frac{p_{i}}{q_{i}},$$

is the Kullback-Leibler divergence between *p* and *q*.

As shown in Kepler et al. [9] Laplace’s integration method with constraints [20] can be used to asymptotically approximate the integral (10) as follows

(13) $$\begin{array}{c} f(p|P,n,d^{-1})={\left\{2\pi \left(\frac{1}{n}+d\right)\right\}}^{-(L_{i}-1)/2}\\ \;\times \overset{L_{i}}{\underset{i=1}{\prod }}\frac{1}{\sqrt{p_{i}}}e^{-\text{nD}(p|q)-d^{-1}D(q|P)} \end{array}$$

and

(14) $$\begin{array}{c} \log f(p|P,n,d^{-1})=-\text{nD}(p|q)-d^{-1}D(q|P)\\ \;-\frac{L_{i}-1}{2}\log\left(\frac{1}{n}+d\right)\\ \;-\frac{L_{i}-1}{2}\log2\pi -\frac{1}{2}\overset{L_{i}}{\underset{i=1}{\sum }}\log p_{i}, \end{array}$$

Moreover, as shown in Kepler et al. [9], a Taylor expansion in *ϵ* = (*nd*)^-1^ of *q*_*i*_ around *p*_*i*_, leads to the following expression for (14)

(15) $$\begin{array}{c} \;\log f(p|P,n,d^{-1})=-d^{-1}\left(D(p|P)-\frac{s_{D}}{2\text{nd}}\right)\\ \;-\frac{L_{i}\;-\;1}{2}\log\left(\frac{1}{n}+d\right)\\ \;-\frac{L_{i}\;-\;1}{2}\log2\pi \;-\;\frac{1}{2}\overset{L_{i}}{\underset{i=1}{\sum }}\log p_{i}\;+\;O(\epsilon^{2}) \end{array}$$

where

(16) $$D(p|P)=\sum p_{i}\log\frac{p_{i}}{P_{i}},$$

and

(17) $$s_{D}=\sum p_{i}{\left(\log\frac{p_{i}}{P_{i}}-D(p|P)\right)}^{2}.$$

From this, one can derive the expected values, *E*, of *D*(*p*|*P*) and *s*_*D*_ up to order *ϵ* to be (for a complete derivation refer to [9])

(18) $$\begin{array}{cc} E\left[D(p|P)\right]=\frac{L_{i}-1}{2}\left(\frac{1}{n}+d\right),\\ \;E\left[s_{D}\right]=(L_{i}-1)\left(d-\frac{1}{n}\right)+O(d\epsilon^{2}). & \end{array}$$

From here we can derive the corrected individual divergence,

(19) $$D_{i,\text{corr}}=D_{f}-\frac{L_{i}-1}{2n},$$

which relaxes the concern of variability due to sampling error.

## Competing interests

The authors declare that they have no competing interests.

## Authors’ contributions

Conceived the study: BHD and TBK. Developed mathematical components: SMC and TBK. Developed empirical components: BHD and MLM. Interpreted results and wrote the manuscript: SMC, BHD, MLM and TBK. All authors read and approved the final manuscript.
